# Non-Negative Symmetric Low-Rank Representation Graph Regularized Method for Cancer Clustering Based on Score Function

**DOI:** 10.3389/fgene.2019.01353

**Published:** 2020-01-22

**Authors:** Conghai Lu, Juan Wang, Jinxing Liu, Chunhou Zheng, Xiangzhen Kong, Xiaofeng Zhang

**Affiliations:** ^1^ School of Information Science and Engineering, Qufu Normal University, Rizhao, China; ^2^ College of Electrical Engineering and Automation, Anhui University, Hefei, China; ^3^ School of Information and Electrical Engineering, Ludong University, Yantai, China

**Keywords:** cancer gene expression data, low-rank representation, feature selection, score function, clustering

## Abstract

As an important approach to cancer classification, cancer sample clustering is of particular importance for cancer research. For high dimensional gene expression data, examining approaches to selecting characteristic genes with high identification for cancer sample clustering is an important research area in the bioinformatics field. In this paper, we propose a novel integrated framework for cancer clustering known as the non-negative symmetric low-rank representation with graph regularization based on score function (NSLRG-S). First, a lowest rank matrix is obtained after NSLRG decomposition. The lowest rank matrix preserves the local data manifold information and the global data structure information of the gene expression data. Second, we construct the Score function based on the lowest rank matrix to weight all of the features of the gene expression data and calculate the score of each feature. Third, we rank the features according to their scores and select the feature genes for cancer sample clustering. Finally, based on selected feature genes, we use the K-means method to cluster the cancer samples. The experiments are conducted on The Cancer Genome Atlas (TCGA) data. Comparative experiments demonstrate that the NSLRG-S framework can significantly improve the clustering performance.

## Introduction

High-throughput DNA microarray technology has long been used to collect biomedical cancer gene expression data (Russo et al., 2003). In general, gene expression data contain a notably large number of genes (high dimension), a small number of samples (low sample size), irrelevant genes and noisy genes caused by complex processing (Mohamad et al., 2010). Therefore, it is necessary to select feature genes or informative genes that contribute to identifying different cancers and the cancerous state (Mohamad et al., 2013; Ge and Hu, 2014; Tang et al., 2014). The selected genes have potential for use in developing cancer treatment strategies (Rappoport and Shamir, 2018). However, the high-dimensional and low-sample-size characteristics of the cancer gene expression dataset present a challenge for researchers in terms of data mining. To mitigate this problem, researchers have proposed many methods (Cui et al., 2013; Ge and Hu, 2014; Wang et al., 2016; Wang et al., 2018; Xu et al., 2019). Among the existing methods, feature selection is a reasonable method that has achieved great success.

Feature selection is an important data processing method that can select the most important feature subset from a set of features and reduce the dimension of the feature space. The existing feature selection methods can be divided into two groups: “wrapper” methods and “filter” methods (Kohavi and John, 1997). Wrapper methods use the learning algorithm to evaluate the candidate features. However, because wrapper methods are highly complex with a large amount of calculation, they are not suitable for large-scale datasets (Langley, 1994). Filter methods select a feature subset via the evaluation function. Construction of an evaluation function is based on the correlations between the features and properties of the raw data, such as the distance measures, information measures, dependence measures or others (Dash and Liu, 1997; Talavera, 2005; He et al., 2006). Among the existing evaluation functions, as a criterion, the data variance might be the simplest evaluation for feature selection. The main idea of the data-variance-based approach is to capture the directions of the maximum variance in the data, which reflects the major power of the data. The Principal Component Analysis (PCA) method and its variants belong to the filter methods and are used to find features that are useful for recovering data. However, there is no reason to confirm that selected features can effectively discriminate between data points in different classes. He et al. proposed the Laplacian Score (LS) method to select features with high identification, and the LS method is a “filter” method that is independent of other methods (He et al., 2006). The LS method constructs a nearest neighbour graph to preserve the local geometric structure. The selected features can reflect the local structure of the data space.

As we know, the global structure plays an important role in clustering when the data contain multiple subspaces (Liu et al., 2010). The LS method focuses excess attention on the relationships between local data points but ignores the influence of global data structures. This drawback might lead to reduced discrimination effects of the selected feature when the given data contain multiple subspaces. For the feature selection method, it is a challenge to satisfactorily characterize and represent global data structures from a dataset with multiple subspaces. Fortunately, the Low-Rank Representation (LRR) method solves this issue nicely. The LRR method can find a low-rank matrix to capture and represent the global structure of the raw dataset (Liu et al., 2010). The key to the LRR method is that the high-dimensional data can be represented by potential low-dimensional subspaces (You et al., 2016). In bioinformatics, LRR has achieved great success in gene expression data mining. For example, Cui et al. used the LRR method to identify subspace gene clusters and obtained good results (Cui et al., 2013). To preserve the intrinsic geometric structures of gene expression data, Wang et al. introduced graph regularization into LRR and proposed the Laplacian regularized LRR (LLRR) method (Wang et al., 2016). Recently, LLRR was applied to cancer sample clustering (Wang et al., 2019a). Furthermore, Wang et al. introduced the mixed-norm to increase the robustness of the LLRR method and proposed the mixed-norm Laplacian regularized LRR (MLLRR) method for tumour sample clustering based on penalized matrix decomposition (Wang et al., 2018). However, cancer sample clustering is processed on the obtained low-rank matrix, which is the global structural representation of the raw data. These LRR-based approaches mainly consider the global structure of data, but sometimes they ignore the single feature gene.

Motivated by the above insights, we propose a novel framework that integrates the advantages of the LRR and LS methods. Based on the multi-cancer gene expression dataset, the proposed framework is used to select the feature gene for cancer sample clustering.

First, we incorporate the constraints of the non-negative symmetric low-rank matrix and graph regularization in the LRR method and propose a non-negative symmetric low-rank representation graph regularized method, or NSLRG method for short. The NSLRG method considers the property and structure of the gene expression data. The NSLRG method obtains the lowest rank matrix, which preserves the local data manifold information and the global data structure information of the raw data.

Second, according to the lowest rank matrix, we construct a Score function to evaluate each gene for selection of the feature genes. The importance level of a gene depends on its significance for the global and local structures of the raw data. We integrate the NSLRG method with the Score function to achieve the aim of evaluating and selecting feature genes, and we refer to it as the NSLRG-S framework.

Finally, we apply the K-means method to cluster cancer samples based on the selected feature genes. Based on the different multi-cancer gene expression data, the experimental results suggest that the performance of the NSLRG-S framework is better than that of other methods.

In summary, the contributions of this paper include the following main aspects:

1. We propose a novel data mining method known as the NSLRG method. The NSLRG method operates under graph regularization and non-negative symmetric low-rank matrix constraints. The NSLRG method can learn the lowest rank matrix to satisfactorily represent the gene expression data and can capture the global structures and local geometric structures of the raw data. Non-negativity is more consistent with biological modelling. The symmetric constraint improves the interpretability of the lowest rank matrix. The constraints of non-negativity and symmetry facilitate the lowest rank matrix to learn the structure of the gene expression data.
2. Based on the lowest rank matrix, we propose a Score function to select the feature genes for cancer sample clustering. The selected feature genes have important significance to the raw data. In the clustering of cancer samples, the selected genes have strong discriminability to realize the classification of different samples.
3. We present a novel feature selection framework, known as NSLRG-S, that is designed to evaluate and select the feature genes for cancer sample clustering. Based on this framework, the selected result of the gene expression dataset has lower dimensionality. In multi-cancer sample clustering, this method has a high recognition rate to find subsets using the selected result as experimental data. We conduct extensive experiments to demonstrate that the feature gene subset selected by NSLRG-S has good performance in cancer sample clustering.

The remainder of this paper is organized as follows. In section *Related Work*, we briefly review the original LRR and several related variants as well as the LS method. In section *Method*, we first present the NSLRG method and its optimal solution, and based on the Score function, the NSLRG-S framework is clearly given for modelling of multi-cancer gene expression data. Section *Experiments* analyses and discusses the NSLRG method based on multiple evaluation indicators and convergence analysis. The performance of the NSLRG-S framework is validated by experiments based on synthetic data and multi-cancer gene expression data. Section *Conclusions Work* presents the conclusion of our work.

## Related Work

In this section, we briefly introduce the original Low-Rank Representation (LRR) (Liu et al., 2010), the related variants based on the original LRR method, and the Laplacian Score method (He et al., 2006).

### Low-Rank Representation

#### Original LRR Method

The Low-Rank Representation (LRR) method is an efficient method for exploring observed data and subspace clustering. The main idea is that each data sample can be represented as a linear combination of the dictionary data. In general, the matrix ***X*** = [*x*_1_,*x*_2_,…,*x*_*n*_]∈*ℝ* ^*m*×*n*^ represents the observed data, of which each column is a data sample. Therefore, the matrix **X** contains *n* data samples drawn from independent subspaces. The matrix ***D*** = [*d*_1_,*d*_2_,…,*d*_*k*_]∈*ℝ* ^*m*×*k*^ represents the dictionary data and is overcomplete. The general model of the LRR method is formulated as follows.

(1) $$\underset{Z}{\min}rank\left(Z\right)\;s.t.\;\;X=DZ,$$

where the matrix ***Z***∈*ℝ* ^*k*×*n*^ is the coefficient matrix. The aim of this model is to learn a lowest rank matrix **Z** ^*^ to represent the observed data **X**. In the actual application, the matrix **X** always replaces **D** as the dictionary data (Liu et al., 2010; Liu et al., 2013). Therefore, **Z** becomes a square matrix and ***Z***∈*ℝ* ^*n*×*n*^. The element ${\text{z}}_{ij}\in Z_{n\times n}^{*}$ can denote the confidence of sample *i* and *j* in the same subspace (Wang et al., 2019b). Hence, the matrix **Z** ^*^ can be used in subspace clustering that clusters data samples into several sets, with each set corresponding to a subspace.

The problem of $\underset{Z}{\min}rank\left(Z\right)$ is a rank function, which is difficult to optimize with an NP-hard nature. To mitigate this problem, the best alternative is convex relaxation on problem (1), and it is written as follows.

(2) $$\underset{Z}{\min}∥Z{∥}_{*}\;s.t.\;\;X=XZ,$$

where ∥⋅∥_*_ is the nuclear norm, and ∥***Z***∥_*_ is defined as $∥Z{∥}_{*}=\sum_{i}^{n}\delta_{i}$, where *δ_i_* is the singular value of matrix ***Z***∈*ℝ* ^*n*×*n*^. It has been confirmed in the literature (Cai et al., 2010) that matrix **Z** of the LRR can capture the global structure of the raw data using the nuclear norm item. Furthermore, to address the real data under the noise and outliers, a more reasonable formula is applied after adjustment, and it is expressed as follows.

(3) $$\underset{Z,E}{\min}∥Z{∥}_{*}+\text{λ}∥E{∥}_{P}\;s.t.\;X=XZ+E,$$

where ∥***E***∥_*P*_ is the error term, and it selects a different *P* to model special noise or outliers based on error prior information, such as *l*_1_-norm (∥***E***∥_1_) and *l*_2,1_- norm (∥***E***∥_2,1_) (Chen and Yang, 2014), and λ > 0 is the parameter that trades off the effect of the error item.

Many researchers have attempted and proposed variants based on the original LRR method. The main idea is to introduce constraint items to optimize or improve existing methods. For example, the original LRR method is improved by considering the geometric structures within the data, including the graph regularization method (Lu et al., 2013) and k-nearest neighbour graph method (Yin et al., 2016). The different norm items are used to improve the robustness of the original LRR method (Wang et al., 2018) and others.

#### LRR With Graph Regularization

Under certain conditions, the geometric structure within the data is crucial for the result that we desire. To address this issue, researchers introduced graph regularization into the LRR method to create the graph-regularized low-rank representation (GLRR) method (Lu et al., 2013). The equation of GLRR is written as follows.

(4) $$\underset{Z,E}{\min}∥Z{∥}_{*}+\lambda_{1}tr\left(ZLZ^{\text{T}}\right)+\lambda_{2}∥E{∥}_{2,1}\;s.t.\;X=XZ+E,$$

where the error item uses the *l*_2,1_-norm and $∥E{∥}_{2,1}=\sum_{j=1}^{n}\sqrt{\sum_{i=1}^{m}{\left({\left[E\right]}_{ij}\right)}^{2}}$, *tr*(⋅) is the trace of the matrix, **L** is the graph Laplacian, and *λ*_1_ and *λ*_2_ are two parameters used to balance the graph-regularized item and the error item. Based on manifold learning, the graph-regularized item achieves the aim that representative data points ***z*** *_i_* and ***z***_j_ can hold the property of the data points *x_i_* and *x_j_* of **X**, which are closed in the intrinsic manifold. Therefore, the inherent geometric structure in the raw data is preserved in the low-rank matrix **Z**.

#### Non-Negative LRR With Sparsity

The non-negativity constraint ensures that every data point is in the convex hull of its neighbours. The sparse constraint ensures that each sample is associated with only a few samples. The non-negative and sparse low-rank matrix supplies a well discriminated weight for the subspace and information group.

Inspired by the above insights, Zhuang et al. proposed the non-negative low rank and sparse graph (NNLRS) method (Zhuang et al., 2012). The formula is given as follows.

(5) $$\underset{Z,E}{\min}∥Z{∥}_{*}+\lambda_{1}∥Z{∥}_{1}+\lambda_{2}∥E{∥}_{2,1}\;s.t.\;X=XZ+E,\;Z>0,$$

where ∥***Z***∥_1_ is the *l*_1_-norm to guarantee the sparsity of coefficient matrix. In real-world applications, the sparsity and non-negativity matrix **Z** obtained by the NNLRS method can offer a basis for semi-supervised learning by constructing the discriminative and informative graph (You et al., 2016).

### Laplacian Score Method

According to the Laplacian eigenmaps (Belkin and Niyogi, 2001) and the locality preserving projection (He and Niyogi, 2005), the aim of the Laplacian Score (LS) method is to evaluate features based on their locality preserving power (He et al., 2006). The LS is defined as follows.

(6) $$LS\left(r\right)=\frac{\sum_{ij}{\left(x_{ri}-x_{rj}\right)}^{2}S_{ij}}{Var\left(x_{r,:}\right)},\;\left(1\leq r\leq m,1\leq i\leq j\leq n\right),$$

where the heat kernel function $S_{ij}=e^{-\frac{∥x_{i}-x_{j}{∥}^{2}}{t}}$ is used to obtain weight matrix *S*, and *t* is a suitable constant, which is set empirically. The matrix *S* is used to model the local structure of the raw data space. Additionally, *Var*(*x*_*r*,:_) is the estimated variance of the *r*-th feature in all data points, and the larger the *Var*(*x*_*r*,:_), the more information held by the *r*-th feature. The $\sum_{ij}{\left(x_{ri}-x_{rj}\right)}^{2}$ is the sum of differences in the expression of *r*-th feature between all samples. For larger values of *S*_ij_ and the smaller values of $\sum_{ij}{\left(x_{ri}-x_{rj}\right)}^{2}$, the value of *LS*(*r*) tends to be smaller, meaning that the importance level of the feature is higher. Therefore, the important features are selected according to *LS*(*r*).

## Method

In this section, we propose a novel feature selection framework to select the feature genes for cancer clustering. This framework is set up based on the NSLRG method and the Score function. We refer to this approach as the NSLRG-S Subsection *NSLRG Method* presents the NSLRG method and its optimization algorithm. In subsection *NSLRG With Score Function*, we introduce the NSLRG method with the Score function. The last subsection *Framework of NSLRG-S* is devoted to clustering of cancer samples based on NSLRG-S modelling of gene expression data.

### NSLRG Method

#### Graph Regularization

Because graph regularization can preserve the intrinsic local geometric structure in the original data, it has received much attention from researchers. The theory of graph regularization is based on the principle that the representation of the intrinsic local geometric structure that is distributed in the original data is inherited by a graph under the new basis mapping. In the graph, the vertices correspond to the data points, and the edge weights represent the relationships between the data points (Du et al., 2017). Thus far, graph theory has been widely applied and developed (Chen et al., 2018).

For this paper, in the step of graph construction, we assume that if data points *x_i_* and *x_j_* are “close”, an edge exists between *x_i_* and *x_j_*. In this work, we use the K-nearest neighbour method to find the connection of *x_i_* and *x_j_*. In other words, if *x_i_* or *x_j_* is among the K-nearest neighbours of each other, the data points *x_i_* and *x_j_* are located on the same edge. This construction strategy is simpler for determination of connected edges, which tends to lead to a connected graph. In the next step, the edge weights are defined to represent the affinity between the data points. In current study, we define a symmetric weighting matrix **W** by the heat kernel weighting function (Cai et al., 2005). The weighting formula is defined as follows.

(7) $$W_{ij}=\{\begin{array}{l} e^{-\frac{∥x_{i}-x_{j}{∥}^{2}}{t}},\;\mathrm{if}x_{i}\mathrm{and}x_{j}\mathrm{are}\mathrm{connected}\\ \;0\;,\;\mathrm{otherwise} \end{array},$$

where the parameter *t* is defined as the mean value of the Euclidean distance for all data points, which can be automatically adjusted based on the different dataset. Therefore, the degree matrix **D** is defined as $D_{ii}=\sum_{j}W_{ij}$, which is a diagonal matrix. Finally, based on the connected graph, we obtain the graph Laplacian matrix **L**, which is defined as follows.

(8) L=D−W.

Accordingly, a reasonable minimize objective function exists to satisfy our assumption, and it is defined as follows.

(9) $$\underset{z}{\min}\sum_{ij}∥z_{i}-z_{j}{∥}^{2}W_{ij}=\underset{z}{\min}tr\left(Z\left(D-W\right)Z^{T}\right)=\underset{Z}{\min}\left(ZLZ^{T}\right),$$

where ***z*** *_i_* and ***z*** *_j_* are mappings of *x_i_* and *x_j_* under the new basis, which are also close to each other if *x_i_* and *x_j_* are close. The objective function is known as the graph regularization item.

#### Objective Function

We introduce graph regularization and sparse items into the original LRR. Furthermore, we impose the non-negative and symmetric constraints on the low-rank matrix **Z**. This method is known as the non-negative symmetric low-rank representation graph regularized (NSLRG) method, and its objective function is written as follows.

(10) $$\underset{Z,E}{\min}∥Z{∥}_{*}+\lambda_{1}tr\left(ZLZ^{\text{T}}\right)+\lambda_{2}∥E{∥}_{1}+\lambda_{3}∥Z{∥}_{0}\;s.t.\;X=XZ+E,\;Z=Z^{T},\;Z>0.$$

In the NSLRG method, we represent a given set of data points as a linear combination of other points using a low-rank matrix **Z**. The low-rank matrix should be sparse to improve the recognition ability. Therefore, the matrix **Z** with a sparse constraint could make the result of the representation more discriminative. However, the ∥***Z***∥_0_ item of problem (10) is NP-hard. Thus, as suggested by matrix completion methods (Candès et al., 2011), we use ∥***Z***∥_1_, a proper relaxed convex item, to replace ∥***Z***∥_0_, and the objective function of NSLRG can be rewritten as follows.

(11) $$\underset{Z,E}{\min}∥Z{∥}_{*}+\lambda_{1}tr\left(ZLZ^{\text{T}}\right)+\lambda_{2}∥E{∥}_{1}+\lambda_{3}∥Z{∥}_{1}\;s.t.\;X=XZ+E,\;Z=Z^{T},\;Z>0.$$

**Figure 1:**
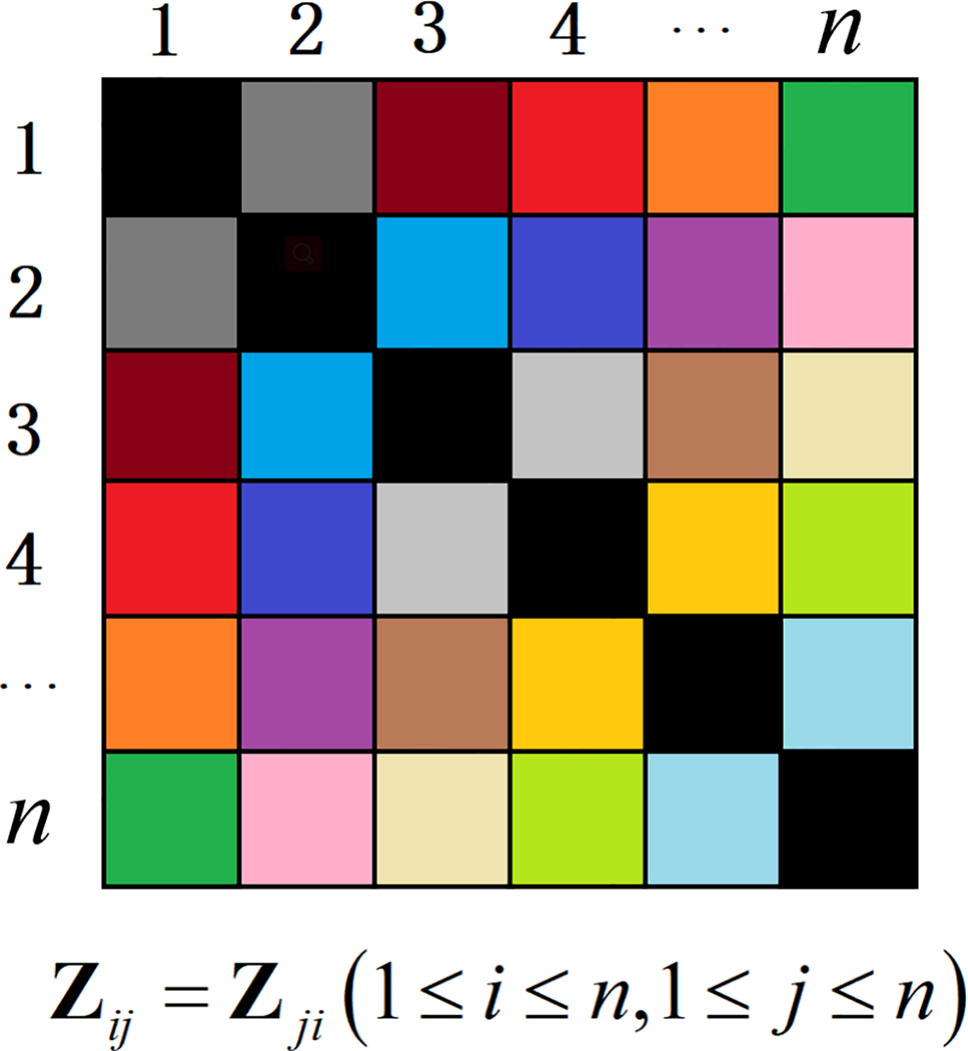
The matrix **Z** with the symmetry constraint.

The matrix **Z** ^*^ is learned by the NSLRG method, and matrix **Z** ^*^ is a non-negative symmetric lowest rank matrix. The element **z** *_ij_* of **Z** ^*^ can be treated as the degree of similarity between the data points *x_i_* and *x_j_*. In addition, the obtained matrix **Z** ^*^ has good interpretability, for which the element of matrix **Z** ^*^ can be directly converted to similar-degree weights. The symmetry constraint can strictly guarantee the consistency of similarity of data pairs. The similarity of data points *i* and *j* corresponding to the similar-degree weights elements **z** *_ij_* and **z** *_ji_* is equal, as shown as **Figure 1**. The non-negative constraint is more adaptive for the property of the gene expression data. In other words, the NSLRG method avoids the situation in which the lowest rank matrix might be negative and asymmetric, and it also avoids symmetrization of itself, as suggested in (Liu et al., 2010), i.e., Z^=(|Z*|+|Z*|T)/2. Therefore, we refer to the matrix **Z** ^*^ as the similar-degree matrix.

#### Optimization

As we know, many algorithms are based on convex relaxation to solve the high-dimension optimization problem, such as Singular Value Thresholding (SVT) (Cai et al., 2010), Accelerated Proximal Gradient (APG) (Toh and Yun, 2010), Alternating Direction Method (ADM) (Lin et al., 2009) and Linearized Alternating Direction Method with Adaptive Penalty (LADMAP) (Lin et al., 2011). As an extended ADM, the LADMAP algorithm adds the quadratic penalty term linearization and the penalty self-adaption change, which leads to use of fewer auxiliary variables and avoids matrix inversions to solve the problem. Specifically, LADMAP reduces the complexity of the LRR from *O*(*n* ^3^) to *O*(*rn* ^3^), where *r* is the rank of low-rank matrix **Z**. This algorithm makes it possible for LRR to be applied on large-scale dataset, such as video surveillance, digital images, and gene expression data. Therefore, the LADMAP algorithm has been recognized as the most efficient algorithm for solving the problem of convex relaxation of low-rank and sparse matrices. Similarly, we also adopt LADMAP to solve (11).

First, to easily and effectively obtain matrix **Z**, we use an auxiliary variable **Q** to separate the variables, i.e., nuclear norm (∥***Z***∥_*_) and *l*_1_-norm (∥***Z***∥_1_). The objective function can be rewritten as equation (12) using the Augmented Lagrange Multiplier method (Lin et al., 2010).

(12) $$\begin{array}{l} \ell \left(Z,E,Q,Y_{1},Y_{2},\mu \right)=\underset{Z,E,Q}{\min}∥Z{∥}_{*}+\lambda_{1}tr\left(ZLZ^{\text{T}}\right)\\ +\lambda_{2}∥E{∥}_{1}+\lambda_{3}∥Q{∥}_{1}+Y_{1},\;X-XZ-E+Y_{2},\;Z-Q\\ +\frac{\mu }{2}∥X-XZ-E{∥}_{F}^{2}+\frac{\mu }{2}∥Z-Q{∥}_{F}^{2}\;s.t.\;\;Z=Z^{\text{T}},Z\geq 0, \end{array}$$

where *λ*_1_, *λ*_2_, and *λ*_3_ are positive weighting parameters; *μ* > 0 is the penalty parameter; **Y**_1_ **_,_Y**_2_ are Lagrangian multipliers; **A**,**B**=*tr*(**A** ^T^ **B**) is the Euclidean inner product between the matrices **A** and **B**; and ∥⋅∥_*F*_ is the Frobenius-norm. Mathematically, equation (12) is equivalent to equation (13) after applying a small transformation. Equation (13) facilitates processing of the next step.

(13) $$\begin{array}{l} \ell \left(Z,E,Q,Y_{1},Y_{2},\mu \right)=\underset{Z,E,Q}{\min}∥Z{∥}_{*}\\ +\lambda_{1}tr\left(ZLZ^{\text{T}}\right)+\lambda_{2}∥E{∥}_{1}+\lambda_{3}∥Q{∥}_{1}\\ +f\left(Z,E,Q,Y_{1},Y_{2},\mu \right)\;\;s.t.\;\;Z=Z^{\text{T}},Z\geq 0. \end{array}$$

Hence, $f\left(Z,E,Q,Y_{1},Y_{2},\mu \right)=\mu \left(∥X-XZ-E+Y_{1}/\mu {∥}_{F}^{2}+∥Z-Q+Y_{2}/\mu {∥}_{F}^{2}\right)/2.$

We divide equation (13) into three subproblems and solve it in three steps. The three subproblems are written as follows.

(14) $$\ell_{1}=\underset{Z}{\min}∥Z{∥}_{*}+\lambda_{1}tr\left(ZLZ^{\text{T}}\right)+f\left(Z,E,Q,Y_{1},Y_{2},\mu \right)\;s.t.\;Z=Z^{\text{T}},Z\geq 0$$

(15) $$\ell_{2}=\underset{E}{\min}\lambda_{2}∥E{∥}_{1}+\mu ∥X-XZ-E+Y_{1}/\mu {∥}_{F}^{2}/2$$

(16) $$\ell_{3}=\underset{Q}{\min}\lambda_{3}∥Q{∥}_{1}+\mu ∥Z-Q+Y_{2}/\mu {∥}_{F}^{2}/2$$

Finally, we solve the above subproblems to find the optimal solution. The specific steps are given as follows.

**Step 1.** Update **Z**: The matrix **Z** can be obtained by solving subproblem *ℓ*_1_ while keeping **E** and **Q** fixed. First, we define the following formula (17) based on *ℓ*_1_.

(17) $$\ell_{1}^{k}\left(Z_{k},E_{k},Q_{k},Y_{1}^{k},Y_{2}^{k},\mu_{k}\right)=\lambda_{1}tr\left(ZLZ^{\text{T}}\right)+f\left(Z_{k},E_{k},Q_{k},Y_{1}^{k},Y_{2}^{k},\mu_{k}\right).$$

By setting the first derivative of $\ell_{1}^{k}$ with respect to **Z** *_k_*, we can obtain the following formula (18).

(18) $$\frac{\partial \ell_{1}^{k}}{\partial Z_{k}}=\lambda_{1}\left(Z_{k}L+Z_{k}L^{\text{T}}\right)+\mu_{k}X^{\text{T}}\left(XZ_{k}-X+E_{k}-Y_{1}^{k}/\mu_{k}\right)+\mu_{k}\left(Z_{k}-Q_{k}+Y_{2}^{k}/\mu_{k}\right).$$

According to LADMAP, subproblem *ℓ*_1_ can be replaced by solving the following problem (19).

(19) $$\underset{Z}{\min}∥Z{∥}_{*}+\frac{\partial \ell_{1}^{k}}{\partial Z_{k}},Z-Z_{k}+\frac{\eta_{1}}{2}∥Z-Z_{k}{∥}_{F}^{2}\;s.t.\;\;Z=Z^{\text{T}},Z\geq 0,$$

where $\eta_{1}=2\lambda_{1}∥L{∥}_{2}+\mu_{k}\left(1+∥X{∥}_{2}^{2}\right)$.

Equation (19) can be transformed into the following formula (20).

(20) $$\underset{Z}{\min}\frac{1}{\eta_{1}}∥Z{∥}_{*}+\frac{1}{2}∥Z-\left(Z_{k}-\frac{\partial \ell_{1}^{k}}{\partial Z_{k}}/\eta_{1}\right){∥}_{F}^{2}\;s.t.\;\;Z=Z^{\text{T}},Z\geq 0.$$

To solve the symmetric and non-negative constraints of low-rank matrix **Z**, we adopt ***Lemma 1*** of (Chen et al., 2017) and the non-negative operator, i.e., equation (24), respectively. ***Lemma 1*** is defined as follows, and the detailed proofs have been given in the literature (Chen et al., 2017).

***Lemma 1***: If there is an expression similar to equation (21), its closed solution is equation (22).

(21) $$\text{arg }\underset{G}{\min}\frac{1}{\beta }∥G{∥}_{*}+\frac{1}{2}∥G-H{∥}_{F}^{2}\;s.t.\;\;G=G^{\text{T}},$$

(22) $$G^{*}=U_{r}\left({\text{Σ}}_{r}-\frac{1}{\beta }I_{r}\right)V_{r}^{\text{T}}.$$

In this work, **U** *_r_*, **∑** *_r_* and **V** *_r_* are the members of the skinny singular value decomposition (SVD) of the matrix $\tilde{G}=U\Sigma V^{\text{T}}$; Σ*_r_* = *diag*(*δ*_1_,*δ*_2_,…,*δ_r_*); *δ_r_* is the singular value for which the positive singular values are greater than $\frac{1}{\beta }$, i.e., $\left\{r:\delta_{r}>\frac{1}{\beta }\right\}$; $\tilde{G}$ is defined as $\tilde{G}=\left(H+H^{\text{T}}\right)/2$; and **I** *_r_* is an identity matrix with size *r* × *r*.

Based on ***Lemma 1***, we make ${\tilde{Z}}_{k}=\frac{1}{2}\left[\left(Z_{k}-\frac{\partial \ell_{1}^{k}}{\partial Z_{k}}/\eta_{1}\right)+{\left(Z_{k}-\frac{\partial \ell_{1}^{k}}{\partial Z_{k}}/\eta_{1}\right)}^{\text{T}}\right]$. We solve the **Z** *_k_*_+1_ using the singular value thresholding operator $\theta_{\epsilon }\left(A\right)=U_{r}S_{\epsilon }\left({\text{Σ}}_{r}-\frac{1}{\eta_{1}}I_{r}\right)V_{r}^{\text{T}}$, where ***S***_*ϵ*_ = sgn(*x*)max(| *x* |−*ϵ*,0). The iterative formula is written as follows.

(23) $$Z_{k+1}=\theta_{\frac{1}{\eta_{1}}}\left(Z_{k}\right),$$

where $\eta_{1}=2\lambda_{1}∥L{∥}_{2}+\mu_{k}\left(1+∥X{∥}_{2}^{2}\right)$. After obtaining matrix **Z** *_k_*_+1_ by equation (23), the non-negative constraint is imposed on matrix **Z** *_k_*_+1_ through a non-negative operator. The non-negative operator is defined as follows.

(24) $$F\left(Z_{k+1}^{*\left(i,j\right)}\right)=\{\begin{array}{c} Z_{k+1}^{\left(i,j\right)},\;\;\;\;\;Z_{k+1}^{\left(i,j\right)}>0\\ 0,\;\;\;\;\;\;\;\;\;\;\;\;\;\;otherwise \end{array}.$$

Finally, the non-negative symmetric low-rank matrix $Z_{k+1}^{*}$ is obtained.

**Step 2.** Update **E**: The matrix **E** can be obtained by solving subproblem *ℓ*_2_ while keeping **Z** and **Q** fixed. Analogously, following equation (18), the first derivative of *ℓ*_2_ is set with respect to **E** *_k_*, i.e., $\frac{\partial \ell_{2}}{\partial E_{k}}$, and set $\frac{\partial \ell_{2}}{\partial E_{k}}=0$. Thus, we obtain equation (25).

(25) $$\frac{\partial \ell_{2}}{\partial E_{k}}=\mu_{k}\left(E_{k}-X+XZ_{k+1}-Y_{1}^{k}/\mu_{k}\right)=0\to E_{k}=X-XZ_{k+1}+Y_{1}^{k}/\mu_{k}.$$

According to the NSHLRR method (Yin et al., 2016), the iterative formula of **E** is given as follows.

(26) $$E_{k+1}={\text{Ψ}}_{\frac{\lambda_{2}}{\mu_{k}}}\left(X-XZ_{k+1}+Y_{1}^{k}/\mu_{k}\right).$$

**Step 3.** Update **Q**: The matrix **Q** can be obtained by solving subproblem *ℓ*_3_ while keeping **Z** and **E** fixed. Similar to **Step 2**, we set the first derivative of *ℓ*_3_ with respect to **Q** *_k_*, i.e.,$\;\frac{\partial \ell_{3}}{\partial Q_{k}}$, and set $\frac{\partial \ell_{3}}{\partial Q_{k}}=0$. Thus, we obtain the following equation.

(27) $$\frac{\partial \ell_{3}}{\partial Q_{k}}=\mu_{k}\left[Q_{k}-\left(Z_{k+1}+Y_{2}^{k}/\mu_{k}\right)\right]=0\to Q_{k}=Z_{k+1}+Y_{2}^{k}/\mu_{k}$$

According to the NSHLRR method (Yin et al., 2016), the iterative formula of **Q** is written as follows.

(28) $$Q_{k+1}=\text{max}\left\{{\text{Ψ}}_{\frac{\lambda_{3}}{\mu_{k}}}\left(Z_{k+1}+Y_{2}^{k}/\mu_{k}\right),0\right\}$$

**Algorithm 1** clearly summarizes the above solution steps. The initialization parameter values are set based on experimental experience and the existing relevant research recommendations (Yin et al., 2016).

**Algorithm 1:** The NSLRG method.

**Input:** data **X**; parameters *λ*_1_, *λ*_2_ and *λ*_3_; the number of *k-*nearest-neighbors.
**Initialization:** $Z_{0}=E_{0}=Q_{0}=Y_{1}^{0}=Y_{2}^{0}=0,$ *ρ*_0_=2.5, *μ*_0_=10^−3^, *μ*_max_=10^6^, *ϵ*_1_=10^−6^, *ϵ*_2_=10^−2^, **L**.
**While not converged do**
1. Update **Z** by **Step1**.
2. Update **E** by **Step2**.
3. Update **Q** by **Step3**.
4. Update Lagrangian multipliers **Y**_1_ and **Y**_2_:
$Y_{1}^{k+1}=Y_{1}^{k}+\mu_{k}\left(X-XZ_{k+1}-E_{k+1}\right)$
$Y_{2}^{k+1}=Y_{2}^{k}+\mu_{k}\left(Z_{k+1}-Q_{k+1}\right)$
5. Update *μ*_*k*+1_:
*μ*_*k*+1_=min(*μ*_max_,*ρ*_*k*_ *μ*_*k*_),
where $\rho_{k}=\{\begin{array}{c} \rho_{0},\;\;if\;max\;\left\{\eta_{1}∥Z_{k+1}-Z_{k}∥,\mu_{k}∥E_{k+1}-E_{k}∥,\;\;\mu_{k}∥Q_{k+1}-Q_{k}∥\right\}\leq \epsilon_{2}\\ 1,\;\;otherwise \end{array}$
**Checking convergence**:
if ||***X***−***XZ***_*k*+1_−***E***_*k*+1_||/||***X***||<*ϵ*_1_ or
max{ *η*_1_∥***Z***_*k*+1_−***Z***_*k*_∥,*μ*_*k*_∥***E***_*k*+1_−***E***_*k*_∥, *μ*_*k*_∥***Q***_*k*+1_−***Q***_*k*_∥ }<*ϵ*_2_
**End while**
**Output:** The lowest rank matrix Z^*^.

### NSLRG With Score Function

It is known that both local structure and global structure can influence the importance of features in raw data. However, the LS method primarily focuses on the locality preserving power of data to evaluate the features. Inspired by the lowest rank matrix Z^*^ of the NSLRG method, which can capture the global and local structure of the raw data, we believe that the important feature of high-dimension data can be extracted based on the matrix Z^*^. Therefore, we propose a Score function that is established on the lowest rank matrix Z^*^ for selection of the important feature. The formula is defined as follows.

(29) $$Score\left(r\right)=\frac{\sum_{ij}{\left(x_{ri}-x_{rj}\right)}^{2}Z_{ij-NSLRG}}{Var\left(x_{r,:}\right)},\;\left(1\leq r\leq m,1\leq i\leq j\leq n\right),$$

where the **Z** *_ij_*_-_ *_NSLRG_* is the element of Z^*^ obtained by the NSLRG method, and **Z** *_ij_*_-_ *_NSLRG_* denotes the similarity degree of the *i*-th and *j*-th samples and is used to measure the *r*-th feature between two samples. The property of the global and local structure captured by the lowest rank matrix can be used as a constraint for feature selection. The selected feature results are quite useful for capturing the subspace structures of raw data. In different classes, this constraint can guarantee the selected feature with high discrimination.

Based on the result of the Score function, all features are arranged in ascending order to form a score curve. The number of selected features is *τ* (*τ* <*m*), which occurs before the first inflection point of the score curve. Thus, we cluster the cancer samples based on the selected feature genes.

We refer to the NSLRG method with the Score function as the NSLRG-S framework for short. In a nutshell, the NSLRG-S framework can be divided into four steps. In the first step, the lowest rank matrix is obtained by the NSLRG method. In the second step, the Score function is used to evaluate and rank features based on the lowest-rank matrix of the first steps. In the third step, the feature genes are selected according to the results of the Score function. In the fourth step, cancer sample clustering is processed based on the selected feature genes. This novel framework delivers better reliability in selection of the most important feature for cancer sample clustering according to the global and local structure of the raw data.

### Framework of NSLRG-S

Based on the proposed NSLRG-S framework, our goal is to model the gene expression data and cluster the cancer samples according to the selected feature genes.

**Figure 2:**
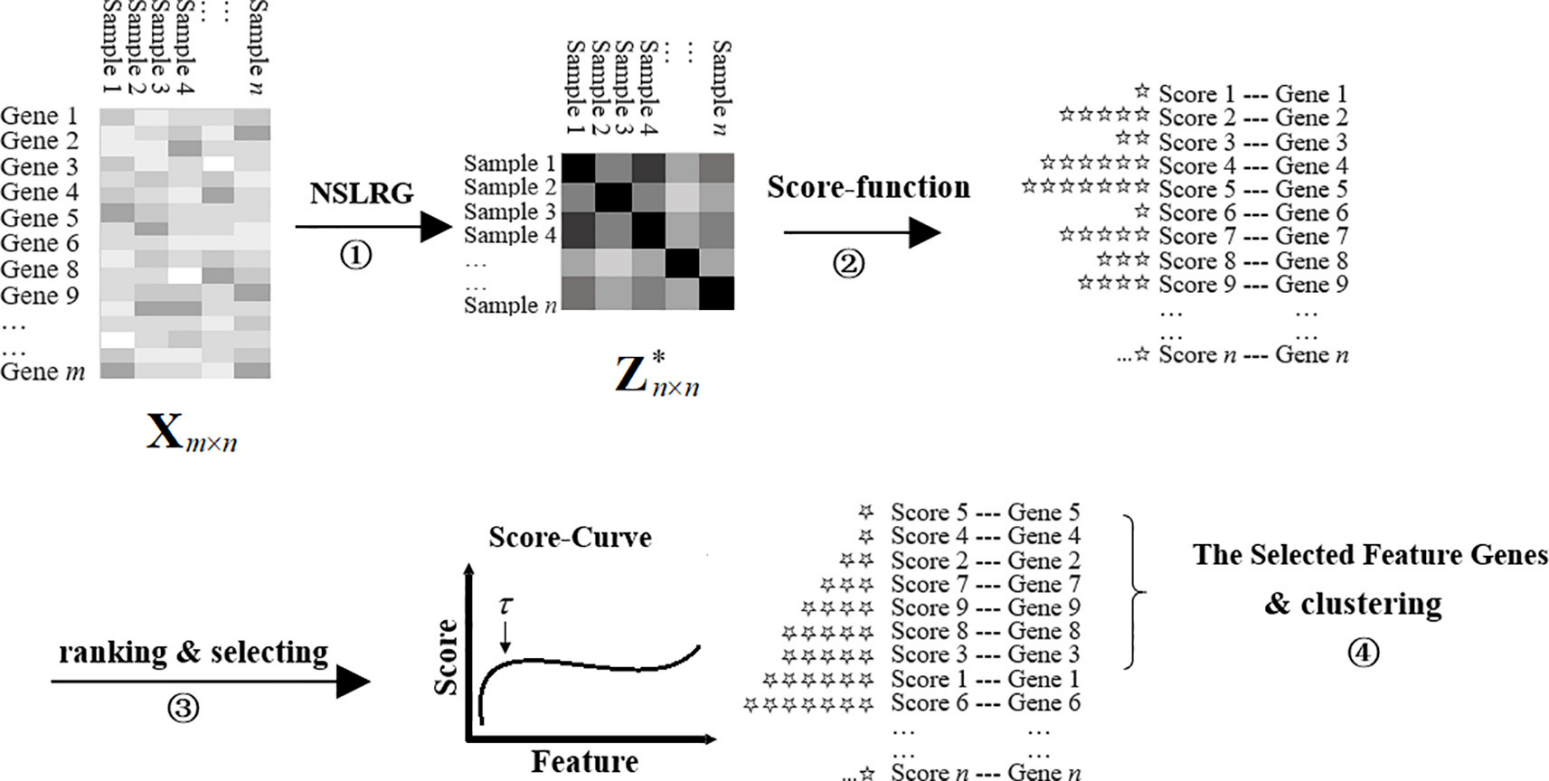
Framework of NSLRG-S for clustering gene expression data.

The modelling process is shown in **Figure 2**. At the start, the matrix **X** *_m_*_×_ *_n_* represents the gene expression data with size *m* × *n*, and one row represents the expression level of a same gene in different samples. The totals of genes and samples are *m* and *n*, respectively. Usually, *m* is notably large and *n* is rather small. The matrix $Z_{n\times n}^{*}$ is the lowest-rank matrix obtained by the NSLRG method as the basis for the Score function. Second, according to the score result, all of the genes are ranked in ascending order. The total number of *τ* (*τ* <*m*) feature genes are selected based on the first inflection point of the score curve. Finally, we cluster the cancer samples based on the selected feature genes to demonstrate the selected genes with efficient discrimination. The result is compared with those of different methods, including the K-means, Graph Regularized Nonnegative Matrix Factorization (GNMF) (Cai et al., 2011), Robust Principal Component analysis (RPCA) (Candès et al., 2011), Sparse Principal Component Analysis (SPCA) (Journée et al., 2010), Graph-Laplacian PCA (GLPCA) (Jiang et al., 2013), LS (He et al., 2006), and LLRR (Wang et al., 2016) methods. The details of the experimental result are described in subsection *Experiments on Gene Expression Data*. **Algorithm 2** is the framework of the NSLRG-S for clustering of gene expression data.

**Algorithm 2:** Framework of NSLRG-S for clustering gene expression data.

**Input:** Gene expression data **X** clustering number *k*
**Step:**
1) Learn a lowest rank matrix Z^*^ by the **Algorithm 1**;
2) Obtain the ranked feature genes by the Score-function;
3) Obtain the selected feature genes.
4) Obtain the clustering cancer samples results using the K-means method.
**Output:** Clustering results

## Experiments

To evaluate the performance of the NSLRG-S framework, we compare the NSLRG-S framework with multiple typical methods, including the K-means, GNMF (Cai et al., 2011), RPCA (Candès et al., 2011), SPCA (Journée et al., 2010), GLPCA (Jiang et al., 2013), LS (He et al., 2006), and LLRR (Wang et al., 2016) methods. In subsection *Evaluation and Quantitative Benchmarks*, we select three quantitative benchmarks to evaluate the experimental results. In subsection *Experiments on Synthetic Data* and subsection *Experiments on Gene Expression Data*, comparative experiments are conducted on synthetic data and cancer gene expression data, respectively.

### Evaluation and Quantitative Benchmarks

To evaluate the performance of the clustering results based on comparison methods, we select three quantitative benchmarks: the clustering accuracy rate (Acc) (Cui et al., 2013), F1 measurement (F1) (Rijsbergen, 1979), and Rand Index (RI) (Rand, 1971).

#### Clustering Accuracy Rate

The Acc is defined as follows.

(30) $$Acc=\frac{\sum_{i=1}^{N}\text{Ξ}\left(\xi_{i},map\left(r_{i}\right)\right)}{N}\times 100\%$$

where *N* is the total number of samples, and Ξ(*ξ*_*i*_,*map*(*r*_*i*_)) is used to identify whether *ξ_i_* and *r_i_* are matched. The *ξ_i_* and *r_i_* are the actual label and clustering label of the *i*-th sample, respectively, and if they are matched, the value of Ξ(*ξ*_*i*_,*map*(*r*_*i*_)) is equal to one; otherwise, its value is equal to zero. The *map*(*r_i_*) is the mapping function based on the Kuhn-Munkres method (Lovász and Plummer, 1986).

#### F1 Measurement

**Figure 3:**
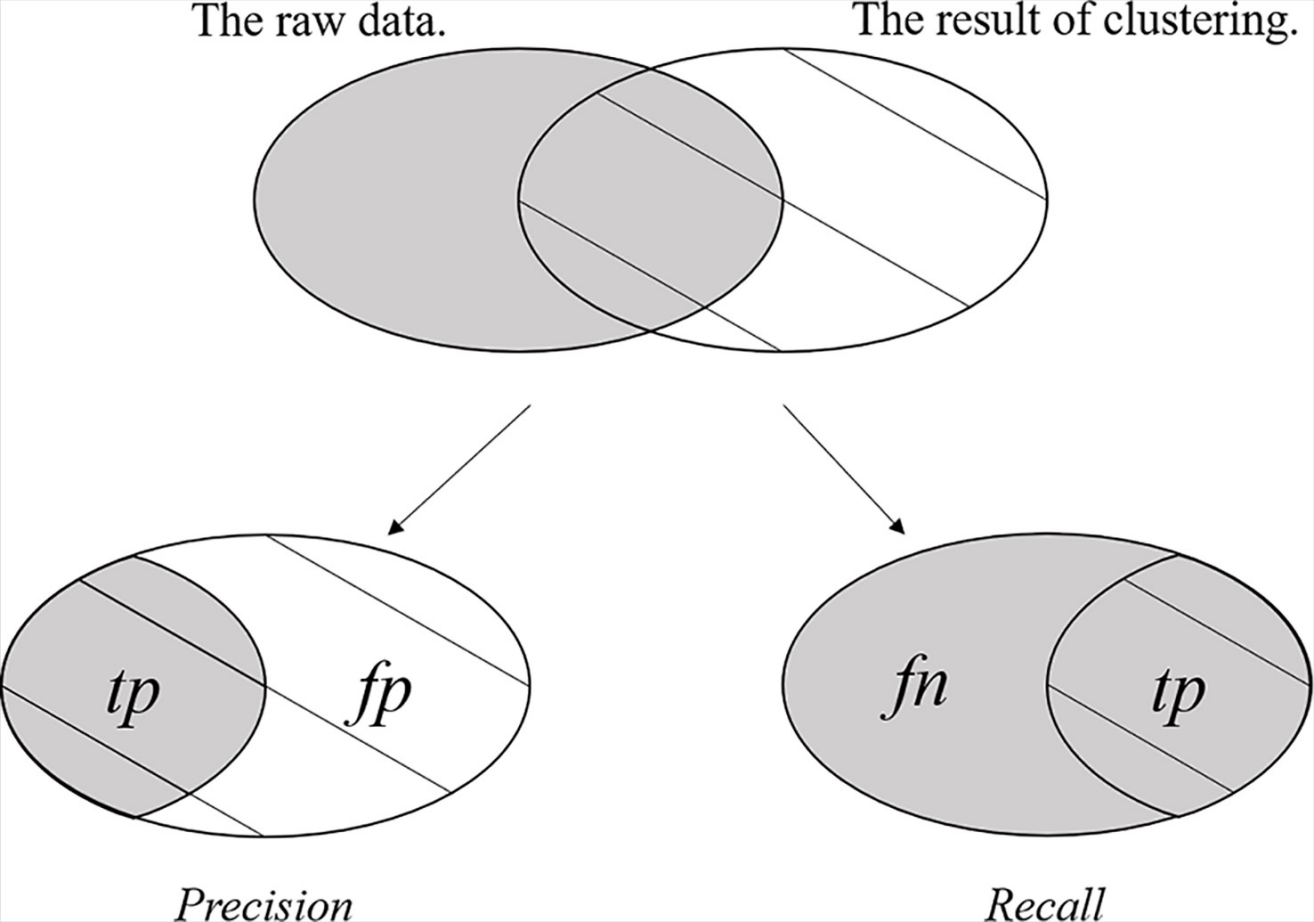
The *tp*, *fp,* and *fn* of the clustering result.

The F1 measurement is a special form of the *F-Measure* under a certain parameter. The *F-Measure* is also referred to as the *F-Score* and is the weighted harmonic mean of the *Precision* rate and *Recall* rate of the result of clustering. The *F-Measure*, *Precision* rate, and *Recall* rate are defined as follows.

(31) $$F=\frac{\left(\phi^{2}+1\right)\times P\times R}{\phi^{2}\times \left(P+R\right)},$$

(32) $$P=\frac{tp}{tp+fp},$$

(33) $$\text{R}=\frac{tp}{tp+fn},$$

where *F* is the *F-Measure*, *P* is the *Precision* rate and *R* is the *Recall* rate. The *tp* (true positives) is the item that records the number of positive samples that are clustered into their own positive class, *fp* (false positives) is the item that records the number of negative samples that are clustered into the positive class, and *fn* (false negatives) is the item that records the number of positive samples that are clustered into negative class. **Figure 3** clearly shows *tp*, *fp* and *fn.* The *F-Measure* can balance the contribution of *fn* by weighting *Recall* through the parameter *ϕ* > 0. When the parameter *ϕ* = 1, *F-Measure* becomes the most common form, i.e., F1 measurement, and equation (31) is rewritten as follows.

(34) $$F1=\frac{2\times P\times R}{P+R}.$$

F1 measurement reaches its best value at 1 and its worst score at 0. The relative contributions of the *Precision* rate and *Recall* rate to the F1 measurement are equal.

#### Rand Index

The given data have two partitions: one is the actual classification, and the other is the clustered result (returned by our **Algorithm 2**). The Rand Index (RI) is used to compute how similar the result of clustering is to the actual classification. The RI is defined as follows.

(35) $$RI=\frac{a+b}{C_{n_{samples}}^{2}},$$

where *a* indicates the number of pairs of data points belonging to the same class in both the actual classification and the clustered result, *b* indicates the number of pairs of data points belonging to the different class in both the actual classification and the clustered result, and $C_{n_{samples}}^{2}$ represents the total number of data pairs obtained from the given data. The range of RI is [0,1], and the larger the value, the more the clustering results are in accordance with reality.

### Experiments on Synthetic Data

In this subsection, comparison experiments are conducted on synthetic data. In subsection *Synthetic Data*, we construct the synthetic data. In subsection *Convergence Analysis*, we perform convergence analysis to compare the NSLRG-S framework and other methods. In subsection *Clustering Results*, we analyze the performance of comparison methods on clustering data samples.

#### Synthetic Data

The synthetic data are constructed by the following steps (1) and (2). These synthetic data contain ten independent subspaces.

1. Construction of 10 original databases by ***O***_*i*+1_ = ***TO***_*i*_, 1 ≤ *i* ≤ 9. The value of the database ranges from 0 to 1, **T** is the transform random rotation matrix, and **O**_1_ is a random orthogonal matrix of 1000×100. The rank of each original database is 100.
2. We extract 10 data vectors from each original database by ***X***_*i*_ = ***O***_*i*_ ***Q***_*i*_,1 ≤ *i* ≤ 10, where the matrix $Q_{i}^{100\times 10}$ is an independent identical distribution matrix *N*(0,1), and its size is 100×10. All extracted data vectors are combined in synthetic data $X_{Synthetic\;data}^{1000\times 100}=\left[X_{1},\;X_{2},\ldots ,X_{10}\right]$.

#### Convergence Analysis

**Figure 4:**
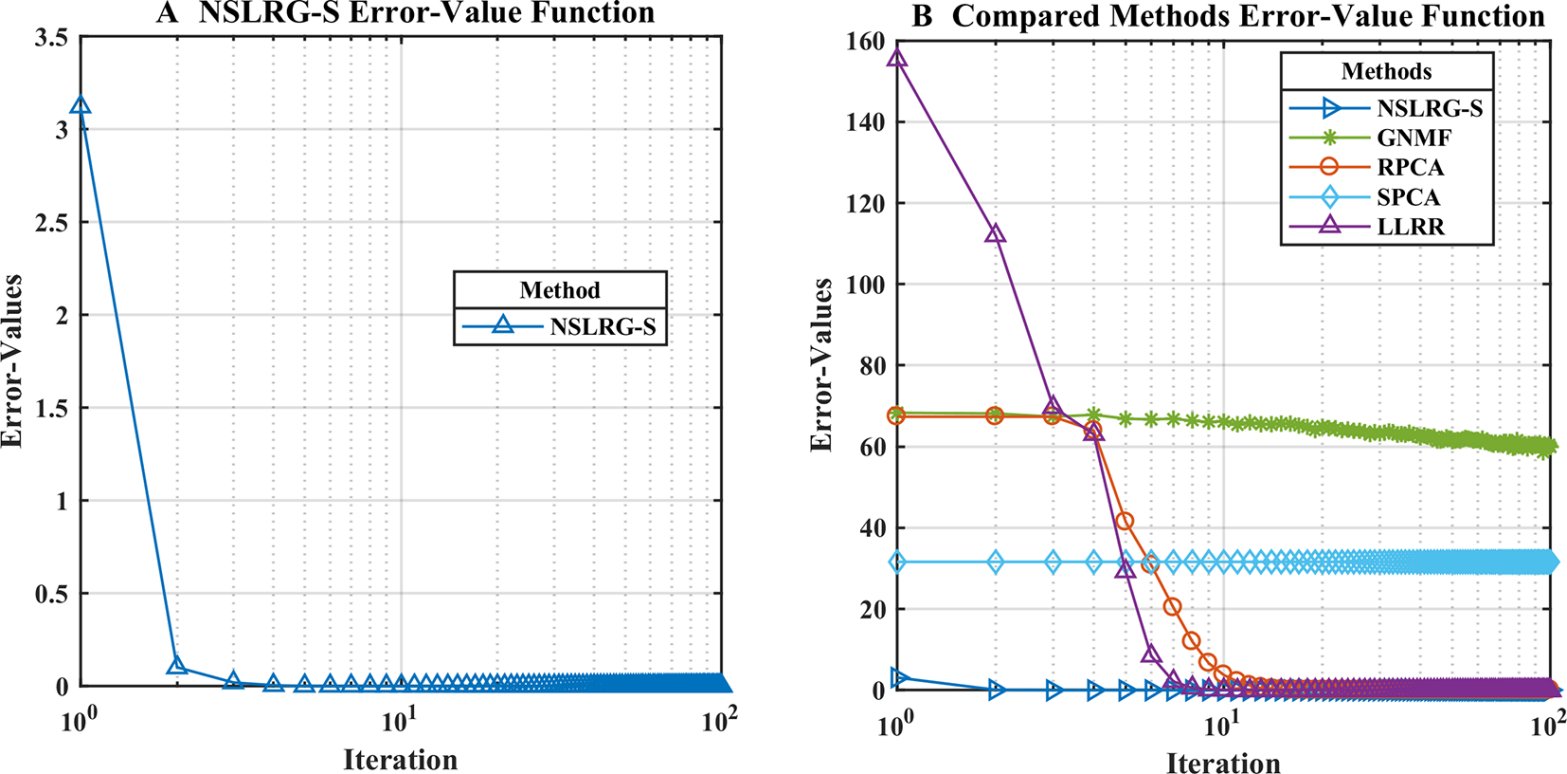
**(A** and **B)**: The convergence analysis of different methods in 100 iterations.

We define an Error-Values function **F** *_E-V_*(*k*) based on the loss function value to calculate the convergence rate. In the same iterations, the smaller the value of the Error-Values, the faster the convergence rate. The formula is given as follows.

(36) $$F_{E-V}\left(k\right)=∥X-\left(XZ_{k}+E_{k}\right){∥}_{F},$$

where the minimum value of **F** *_E-V_*(*k*) is equal to zero. To clearly characterize the convergence rate, **Figures 4A, B** show the convergence trends of the NSLRG-S and the compared methods GNMF, RPCA, SPCA, and LLRR in 100 iterations. In **Figure 4B**, we find that the convergence rate of the NSLRG method is faster than those of the other methods.

#### Clustering Results

**Table 1** lists the results of the GNMF, RPCA, SPCA, GLPCA, LS, LLRR, and NSLRG-S methods on the three quantitative benchmarks as Acc, F1, and RI. The results show that the performance of NSLRG-S is better than those of other methods.

**Table 1:** The clustering results of compared methods and NSLRG-S method on synthetic data.

Method	Acc (%)	F1 (%)	RI (%)
GNMF	72.44	68.42	93.01
RPCA	80.68	78.82	95.57
SPCA	70.42	67.6	91.07
GLPCA	67.28	64.45	89.84
LS	80.62	78.37	96.12
LLRR	81.04	78.67	96.12
NSLRG-S	**82.00**	**79.21**	**96.27**

Acc, clustering accuracy rate; F1, F1 measurement; and RI, Rand Index; GNMF, Graph Regularized Nonnegative Matrix Factorization; SPCA, Sparse Principal Component Analysis; GLPCA, Graph-Laplacian PCA; LS, Laplacian Score; and LLRR, Laplacian regularized Low-Rank Representation; NSLRG-S, non-negative symmetric low-rank representation with graph regularization based on score function. The bolded texts mean the results are better than the others.

### Experiments on Gene Expression Data

In this subsection, we conduct experiments on gene expression datasets. The experimental datasets are downloaded from the famous gene expression database The Cancer Genome Atlas (TCGA). We cluster the cancer samples based on the feature genes obtained by the NSLRG-S framework. The experimental results demonstrate that we can improve the performance in cancer samples clustering by applying the selected feature genes.

#### Gene Expression Datasets

The TCGA database is a source of experimental data and is an important project for accelerating and comprehensively understanding cancer genetics using innovative genome analysis technologies (Tomczak et al., 2015). This database is one of the invaluable sources for gene expression datasets. Therefore, we select the TCGA database as the data source to research the clustering performance of the NSLRG-S framework.

We downloaded five cancer gene expression datasets, namely, esophageal carcinoma (ESCA), head and neck squamous cell carcinoma (HNSC), cholangiocarcinoma (CHOL), colon adenocarcinoma (COAD) and pancreatic adenocarcinoma (PAAD). Each type of gene expression dataset contains cancer tissue samples and normal tissue samples. There are 20,502 genes in each tissue sample. The distribution of each gene expression dataset is listed in **Table 2**.

**Table 2:** The distribution of five gene expression datasets.

Dataset	Cancer tissue samples	Normal tissue samples	Total samples	Total genes
PAAD	176	4	180	20502
HNSC	398	20	418	20502
ESCA	183	9	192	20502
COAD	262	19	281	20502
CHOL	36	9	45	20502

In addition, to find the feature gene with a high recognition rate between different cancers for cancer sample clustering, we construct seven mixed datasets. The mixed datasets are HN-PA, ES-PA, CO-ES and HN-CH; HN-PA-CH, ES-PA-CH, and CO-PA-CH. The construction rule combines tumour tissue samples that come from different gene expression data, and the combined datasets contain two or three types of cancers. For example, in the HN-PA data, HN represents all of the cancer tissue samples of the HNSC data, and PA represents the total of the cancer tissue samples of the PAAD data. The cancer tissue samples of HN and PA are combined to construct the new mixed data, i.e., HN-PA, which contain two types of cancers and have 574 cancer tissue samples. For the other mixed datasets, the distributions are listed in **Table 3**.

**Table 3:** The distribution of mixed datasets.

Dataset	Cancer tissue and the number	Total number
HN-PA	398 from HNSC; 176 from PAAD;	574
ES-PA	183 from ESCA; 176 from PAAD;	359
CO-ES	262 from COAD; 183 from ESCA;	445
HN-CH	398 from HNSC; 36 from CHOL;	434
HN-PA-CH	398 from HNSC; 176 from PAAD; 36 from CHOL;	610
ES-PA-CH	183 from ESCA; 176 from PAAD; 36 from CHOL;	395
CO-PA-CH	262 from COAD; 176 from PAAD; 36 from CHOL;	474

ESCA, esophageal carcinoma; HNSC, head and neck squamous cell carcinoma; CHOL, cholangiocarcinoma; COAD, colon adenocarcinoma; and PAAD, pancreatic adenocarcinoma.

The five original datasets and seven mixed datasets are used in experiments. We classify all datasets into three categories according to the number of cancers they contain. The datasets that contain one type of cancer belong to Category I. Thus, Category I contains PAAD, HNSC, ESCA, COAD, and CHOL. Datasets that contain two types of cancers belong to Category II, and they are HN-PA, ES-PA, CO-ES, and HN-CH. The datasets that contain three types of cancers belong to Category III, and the names of these datasets are HN-PA-CH, ES-PA-CH, and CO-PA-CH. **Table 4** clearly lists the category results.

**Table 4:** The category result of experimental datasets.

Category	I	II	III
Dataset	PAAD	HN-PA	HN-PA-CH
	HNSC	ES-PA	ES-PA-CH
	ESCA	CO-ES	CO-PA-CH
	COAD	HN-CH	/
	CHOL	/	/

ESCA, esophageal carcinoma; HNSC, head and neck squamous cell carcinoma; CHOL, cholangiocarcinoma; COAD, colon adenocarcinoma; and PAAD, pancreatic adenocarcinoma.

#### Parameter Selection

In the experiments, we need to select the optimal parameters of the different datasets. For the three parameters (*λ*_1_, *λ*_2_, *λ*_3_) of the NSLRG method, we assume that the optimal value of each parameter exists within an estimation range of 10^*t*^(*t* = { −5,−4,−3,−2,−1,0,1,2,3,4,5 }). We study the influence of each parameter on feature selection and select the optimal parameters according to the different datasets. First, our main task is to determine the sensitivity of each parameter to the different datasets. We change one parameter within the candidate interval while holding the other two parameters fixed to explore the influence degree of this parameter on the dataset. We find that the parameter *λ*_3_ is insensitive for all datasets. Therefore, the NSLRG method is robust for the parameter *λ*_3_, and we select the *λ*_3_ = 10^-3^ according to experimental experience. The details of selection of the other two parameters are listed in **Table 5**.

**Table 5:** The parameter selection.

Dataset	*λ*_1_	*λ*_2_	*λ*_3_
PAAD	10^-5^	10^-2^	10^-3^
HNSC	10^-3^	10^-4^	10^-3^
ESCA	10^4^	10^-1^	10^-3^
COAD	10^4^	10^0^	10^-3^
CHOL	10^-1^	10^-1^	10^-3^
HN-PA	10^-4^	10^1^	10^-3^
ES-PA	10^-2^	10^-1^	10^-3^
CO-ES	10^2^	10^5^	10^-3^
HN-CH	10^-1^	10^5^	10^-3^
HN-PA-CH	10^-5^	10^-2^	10^-3^
ES-PA-CH	10^-4^	10^0^	10^-3^
CO-PA-CH	10^1^	10^-2^	10^-3^

ESCA, esophageal carcinoma; HNSC, head and neck squamous cell carcinoma; CHOL, cholangiocarcinoma; COAD, colon adenocarcinoma; and PAAD, pancreatic adenocarcinoma.

#### Results and Discussion

In this subsection, based on the datasets of subsection *Gene Expression Datasets*, we apply the NSLRG-S to cluster the cancer samples. We adopt seven clustering methods, including K-means, GNMF, RPCA, SPCA, GLPCA, LS, and LLRR, for comparison with NSLRG-S.

Typically, gene expression data mining can be recognized as addressing a small sample size and high-dimensional problem. The applied methods must face and suffer from what is known as the curse of dimensionality. This situation occurs because the more dimensions contained in the data (20,502 in our case), the more unstable the result. Therefore, in our experiments, we improve the reasonableness of the result by running the experiment 50 times. The mean of the results is taken as the measurement of the clustering results.

**Figure 5:**
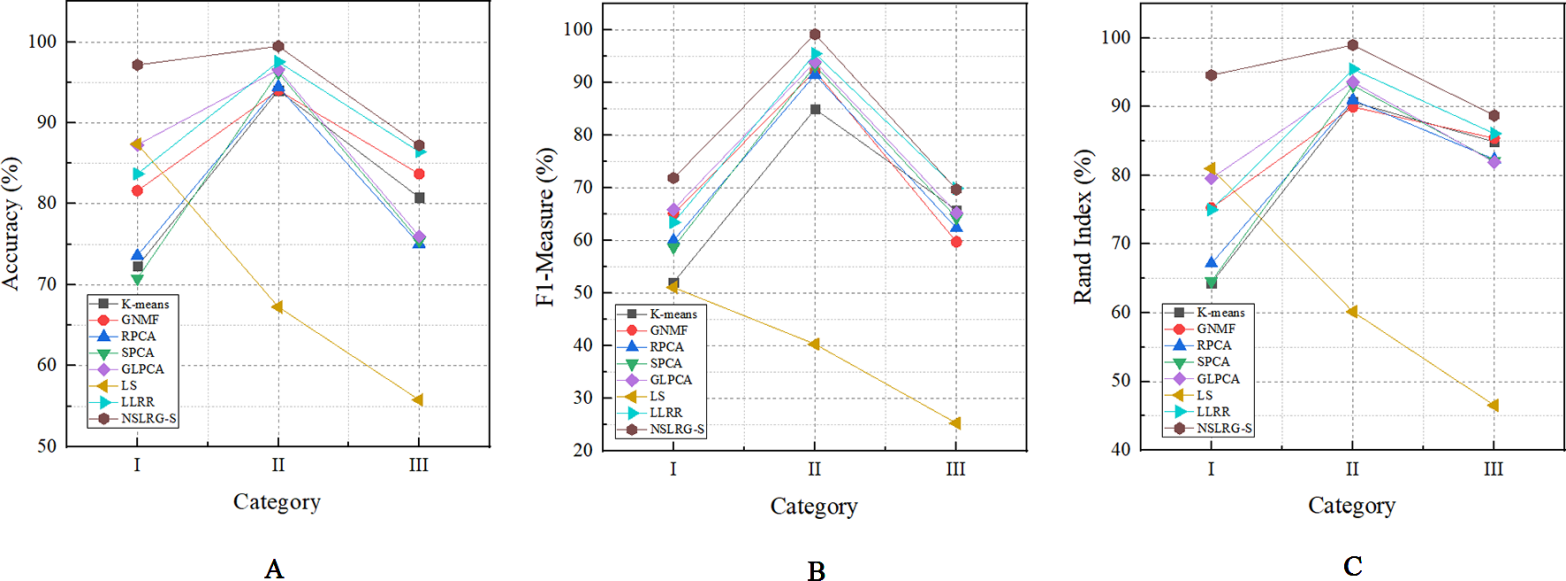
The mean metrics of experimental result for Category I, II, and III. **(A)** Accuracy-Category **(B)** F1-Category **(C)** Rand Index-Category.

**Figure 6:**
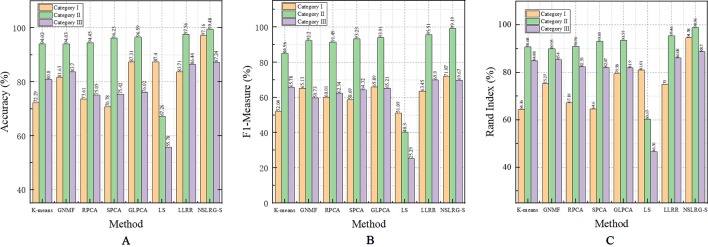
The mean metrics of experimental result for all methods. **(A)** Accuracy-Method **(B)** F1-Method **(C)** Rand Index-Method.

**Table 6** clearly lists the experimental results of all methods. Based on **Table 6**, we obtain the mean metrics of each category dataset, and they are listed in **Table 7**. Furthermore, to clearly show the experimental results on different categories of dataset and different methods, **Figure 5** presents a broken-line graph for the three category datasets corresponding to different methods. **Figure 6** presents a histogram for the different methods corresponding to the three category datasets.

**Table 6:** The result of comparison experiment.

Category	Dataset	Measure	K-means	GNMF	RPCA	SPCA	GLPCA	LS	LLRR	NSLRG-S
I	PAAD	Acc	69.50%	74.67%	63.49%	56.47%	76.53%	**97.78%**	81.46%	97.22%
		F1	43.28%	46.69%	41.42%	40.31%	45.53%	**66.10%**	48.45%	49.30%
		RI	63.77%	61.96%	55.23%	50.58%	64.45%	**95.63%**	69.73%	94.57%
	HNSC	Acc	69.50%	81.72%	64.52%	62.20%	90.71%	93.54%	81.44%	**94.37%**
		F1	46.78%	44.97%	47.34%	46.59%	68.51%	48.33%	48.43%	**48.55%**
		RI	59.44%	70.05%	54.19%	52.86%	83.68%	87.89%	69.69%	**89.36%**
	ESCA	Acc	62.01%	54.69%	53.65%	53.97%	84.90%	94.79%	67.47%	**94.91%**
		F1	43.97%	40.00%	40.22%	41.15%	46.74%	48.66%	46.97%	**64.18%**
		RI	58.34%	50.18%	50.01%	50.06%	76.19%	90.07%	56.41%	**90.40%**
	COAD	Acc	74.71%	**99.29%**	86.39%	81.28%	84.42%	87.09%	88.20%	**99.29%**
		F1	60.02%	**97.31%**	71.08%	65.41%	68.68%	47.54%	73.40%	**97.31%**
		RI	65.22%	**98.58%**	76.45%	69.48%	73.60%	78.08%	79.15%	**98.58%**
	CHOL	Acc	85.72%	97.78%	**100.00%**	**100.00%**	**100.00%**	63.82%	**100.00%**	**100.00%**
		F1	66.16%	96.66%	**100.00%**	**100.00%**	**100.00%**	44.81%	**100.00%**	**100.00%**
		RI	75.03%	95.56%	**100.00%**	**100.00%**	**100.00%**	53.36%	**100.00%**	**100.00%**
II	HN-PA	Acc	97.66%	99.83%	99.48%	99.30%	98.95%	68.95%	99.65%	**100.00%**
		F1	95.99%	99.80%	99.39%	99.19%	98.78%	41.77%	99.59%	**100.00%**
		RI	96.38%	99.65%	98.96%	98.61%	97.93%	57.11%	99.30%	**100.00%**
	HN-CH	Acc	85.42%	98.39%	82.56%	89.59%	92.06%	90.12%	94.14%	**99.54%**
		F1	73.89%	94.18%	71.16%	77.83%	81.62%	47.40%	86.08%	**98.45%**
		RI	76.94%	96.82%	72.33%	81.36%	85.37%	82.15%	89.46%	**99.08%**
	ES-PA	Acc	96.41%	97.21%	98.25%	99.16%	99.16%	50.86%	99.16%	**99.72%**
		F1	73.89%	97.21%	97.95%	99.16%	99.16%	34.37%	99.16%	**99.72%**
		RI	95.44%	94.57%	97.37%	98.34%	98.34%	49.89%	98.34%	**99.44%**
	CO-ES	Acc	96.58%	80.67%	97.53%	96.85%	96.18%	59.10%	97.30%	**98.65%**
		F1	96.07%	77.59%	97.45%	96.75%	96.06%	37.65%	97.21%	**98.60%**
		RI	93.95%	68.75%	95.17%	93.89%	92.63%	51.55%	94.74%	**97.33%**
III	HN-PA-CH	Acc	81.01%	**92.79%**	77.20%	78.83%	80.13%	65.25%	87.71%	**88.62%**
		F1	62.79%	63.16%	61.82%	63.15%	65.25%	26.69%	**70.03%**	63.36%
		RI	84.14%	**94.79%**	81.99%	81.85%	81.76%	51.20%	87.74%	89.98%
	ES-PA-CH	Acc	81.14%	68.86%	73.91%	72.78%	72.52%	46.51%	86.03%	**89.37%**
		F1	65.98%	52.42%	63.41%	66.55%	66.13%	22.30%	69.23%	**72.11%**
		RI	86.29%	77.41%	82.73%	80.33%	80.29%	42.64%	85.98%	**90.58%**
	CO-PA-CH	Acc	80.24%	**89.45%**	74.04%	74.63%	75.40%	55.59%	85.57%	83.74%
		F1	68.56%	63.60%	61.77%	63.27%	64.27%	26.89%	70.44%	**73.56%**
		RI	84.22%	84.00%	82.27%	84.02%	83.65%	45.84%	84.53%	**85.52%**

ESCA, esophageal carcinoma; HNSC, head and neck squamous cell carcinoma; CHOL, cholangiocarcinoma; COAD, colon adenocarcinoma; and PAAD, pancreatic adenocarcinoma. The bolded texts mean the results are better than the others.

**Table 7:** The mean metrics of result for all methods on Category dataset I, II, III.

Metrics	Category	K-means	GNMF	RPCA	SPCA	GLPCA	LS	LLRR	NSLRG-S
ACC	I	72.29%	81.63%	73.61%	70.78%	87.31%	87.40%	83.71%	**97.16%**
	II	94.02%	94.03%	94.45%	96.23%	96.59%	67.26%	97.56%	**99.48%**
	III	80.80%	83.70%	75.05%	75.42%	76.02%	55.78%	86.44%	**87.24%**
F1	I	52.04%	65.13%	60.01%	58.69%	65.89%	51.09%	63.45%	**71.87%**
	II	84.96%	92.20%	91.49%	93.23%	93.91%	40.30%	95.51%	**99.19%**
	III	65.78%	59.73%	62.34%	64.32%	65.21%	25.29%	**69.90%**	69.67%
RI	I	64.36%	75.27%	67.18%	64.60%	79.58%	81.01%	75.00%	**94.58%**
	II	90.68%	89.95%	90.96%	93.05%	93.57%	60.17%	95.46%	**98.96%**
	III	84.88%	85.40%	82.33%	82.07%	81.90%	46.56%	86.08%	**88.70%**

Acc, clustering accuracy rate; F1, F1 measurement; and RI, Rand Index; GNMF, Graph Regularized Nonnegative Matrix Factorization; SPCA, Sparse Principal Component Analysis; GLPCA, Graph-Laplacian PCA; LS, Laplacian Score; and LLRR, Laplacian regularized Low-Rank Representation; NSLRG-S, non-negative symmetric low-rank representation with graph regularization based on score function. The bolded texts mean the results are better than the others.

By comparing the clustering results of NSLRG-S and other methods, we find that the results of the NSLRG-S method are the best of all methods in most datasets. According to **Table 6**, for the Category I dataset, the clustering performance of NSLRG-S for the HNSC and ESCA datasets is higher than that of other methods. In the COAD and CHOL dataset, NSLRG-S achieves the same best results as the other methods. For the Category II dataset, the clustering performance of NSLRG-S is the best of all methods. For the Category III dataset, except for the metrics of Acc and F1 on HN-PA-CH and Acc on CO-PA-CH, which are obtained by GNMF, and F1 on HN-PA-CH obtained by LLRR, the clustering performance of NSLRG-S is better than that of other methods.

In addition to the numerical comparison, we also find that the NSLRG-S method has different advantages after comparing it with different comparison methods. In the next section, we conduct a more detailed comparison and analysis between NSLRG-S and the other comparison methods.

In the seven comparison methods (K-means, GNMF, RPCA, SPCA, GLPCA, LS, and LLRR), K-means is the traditional clustering method; GNMF belongs to matrix factorization techniques, which extend the nonnegative matrix factorization with preservation of the intrinsic geometric structure (Cai et al., 2011); RPCA, SPCA, and GLPCA are variant methods of principal component analysis, which is a well-established descending dimension method for mining high dimensional data (Journée et al., 2010); LS is the feature selection method; and the LLRR is the subspace clustering method. In addition, the NSLRG-S framework combines the NSLRG method and Score function. Therefore, this framework belongs to a mixed method that combines the advantage of both sides.

First, we compare the NSLRG-S framework with K-means. Based on **Table 6**, we find that a higher clustering result is obtained by NSLRG-S. This comparison result shows that the proposed NSLRG-S framework is better than the traditional clustering method in cancer sample clustering. This result occurs because the NSLRG-S considers the local and global structure of the raw data. This framework can select feature genes with a high recognition rate for cancer sample clustering. In addition, the K-means method performs cancer sample clustering based on the raw data, which ignores the contents considered in NSLRG-S. **Figure 5** clearly shows that the NSLRG-S is superior to the K-means method.

Second, we compare the NSLRG-S with the GNMF method. In GNMF, a nearest neighbour graph is constructed by encoding the geometrical information of the data space. The method seeks matrix factorization, which incorporates the graph structure (Cai et al., 2011). Based on **Table 5**, the GNMF method obtains good results, and a subset of them are even better than those of NSLRG-S method. For most of the datasets, the results of NSLRG-S are still better than those of GNMF. The reason for this result is that the NSLRG-S method can obtain the characteristics of the subspace structure of the raw data, and the corresponding subspace of different types of cancer can be satisfactorily distinguished.

Third, we compare the NSLRG-S with the RPCA, SPCA, and GLPCA methods. RPCA, SPCA, and GLPCA belong to principal component analysis methods and are suitable for processing high-dimensional gene expression data by learning a low-dimensional representation. The results of NSLRG-S are better than those of three methods, except for the CHOL dataset. We can conclude that the NSLRG-S method is better than the variant methods of principal component analysis in clustering of multiple cancer samples.

Fourth, we compare the NSLRG-S with the LS method. Based on **Figure 5**, we find that the performance of LS decreases gradually on the Category I, Category II and Category III datasets, and this trend is different with other methods. The reason for this result is that the feature genes selected by the LS method have locality-preserving power attributes but do not have good multi-subspace separation attributes. In the framework of the NSLRG-S, feature genes are obtained under the Score function based on the low-rank matrix obtained by the NSLRG method. This low-rank matrix can preserve the global and local structure of the raw data, and after further processing the low-rank matrix through the Score function, the selected genes have a strong discrimination in multi-subspace clustering. Therefore, the performance of NSLRG-S is better than that of LS.

Finally, we compare the NSLRG-S with the LLRR method. Based on **Figure 5**, the broken line of the NSLRG-S is always above that of the LLRR method except for F1 on the Category III dataset. The comparison results show that the Score function plays an important role in further mining of the low-rank matrix of the NSLRG method.

Furthermore, we note an interesting trend in the results of three categories of datasets for each method, as shown in **Figure 6**. Other than the LS method, which shows a downward trend, the other methods show an upward trend first followed by a downward trend. In other words, except for the LS method, after comparing all of the results of the other methods, we note that the experimental results of the Category II datasets are the best, followed by the Category III datasets or the Category I datasets, and this trend occurs in all metrics. According to **Tables 2**–**4**, the distributions of sample size in the Category II datasets are more balanced than those in Category I and Category III. Therefore, the result of the Category II dataset is more reasonable and stable than the results of Category I and Category III. However, with an increasing number of subspaces, the structure of the data is more complex, and the global and local structures of raw data are more difficult to capture. Therefore, compared with the experimental results of the Category II datasets, the experimental results of the Category III datasets decrease. Fortunately, according to **Table 7**, the NSLRG-S is still better than other methods. This observation demonstrates that the NSLRG-S framework has better advantages in cancer sample clustering than other methods when working with unbalanced and multi-subspace datasets. Based on the above discussion and analysis, we conclude that the NSLRG-S framework has a good effect for cancer sample clustering based on a gene expression dataset.

## Conclusions Work

In this paper, we cluster the cancer samples of multi-cancer gene expression datasets based on select feature genes obtained by the NSLRG-S framework. In addition, NSLRG-S simultaneously considers the local and global structure of the raw gene expression dataset. The selected feature genes have a high recognition rate in subspace clustering. The comparison experimental results suggest that the NSLRG-S framework can significantly improve the cancer samples clustering performance.

## Data Availability Statement

The datasets generated for this study can be found in the [The Cancer Genome Atlas (TCGA)] https://cancergenome.nih.gov/. We have uploaded scripts and examples on GitHub to adhere standards for reproducibility. The URL is https://github.com/guoguoguolu/NSLRG-S-method-scripts-and-example-files.

## Author Contributions

JW and CL conceived the original research plans and methodology. JL and XK performed synthetic data analysis. JW, CL and XK performed experiments on gene expression data. JW and CL supervised and wrote the original draft. JW, CZ, and XZ reviewed and revised the writing.

## Funding

This work was supported in part by the National Natural Science Foundation of China under Grant Nos. 61872220, 61702299, and 61873117.

## Conflict of Interest

The authors declare that the research was conducted in the absence of any commercial or financial relationships that could be construed as a potential conflict of interest.
